# Genetic variations for the eggshell crystal structure revealed by genome-wide association study in chickens

**DOI:** 10.1186/s12864-021-08103-1

**Published:** 2021-11-02

**Authors:** Quanlin Li, Zhongyi Duan, Congjiao Sun, Jiangxia Zheng, Guiyun Xu, Ning Yang

**Affiliations:** 1 National Engineering Laboratory for Animal Breeding and MOA Key Laboratory of Animal Genetics and Breeding, Department of Animal Genetics and Breeding, China Agricultural University, 100193 Beijing, China; 2 National Animal Husbandry Service, 100125 Beijing, China

**Keywords:** Chicken, Eggshell, Crystal structure, X-ray diffraction, GWAS

## Abstract

**Background:**

Eggshell is a bio-ceramic material comprising columnar calcite (CaCO_3_) crystals and organic proteinaceous matrix. The size, shape and orientation of the CaCO_3_ crystals influence the microstructural properties of chicken eggshells. However, the genetic architecture underlying eggshell crystal polymorphism remains to be elucidated.

**Results:**

The integral intensity of the nine major diffraction peaks, total integral intensity and degree of orientation of the crystals were measured followed by a genome-wide association study in 839 F2 hens. The results showed that the total integral intensity was positively correlated with the eggshell strength, eggshell thickness, eggshell weight, mammillary layer thickness and effective layer thickness. The SNP-based heritabilities of total integral intensity and degree of orientation were 0.23 and 0.06, respectively. The 621 SNPs located in the range from 55.6 to 69.1 Mb in GGA1 were significantly associated with TA. *PLCZ1*, *ABCC9*, *ITPR2*, *KCNJ8*, *CACNA1C* and *IAPP*, which are involved in the biological process of regulating cytosolic calcium ion concentration, can be suggested as key genes regulating the total integral intensity.

**Conclusions:**

The findings greatly advance the understanding of the genetic basis underlying the crystal ultrastructure of eggshell quality and thus will have practical significance in breeding programs for improving eggshell quality.

**Supplementary Information:**

The online version contains supplementary material available at 10.1186/s12864-021-08103-1.

## Background

In the global egg industry, the integrity of the eggshell is critical for the economic viability of poultry production. The shells must be strong enough to prevent failure during packing and transportation [1]. The eggshell also forms an embryonic chamber for the developing chick by providing mechanical protection, allowing gas exchange and preventing microbial contamination [2, 3]. The process of eggshell formation is also a classic model for studying biomineralization [4]. The avian eggshell is composed of calcium carbonate, organic matrix and other minerals [5–7]. Several factors have been shown to affect eggshell structure [5, 8, 9] with most studies still based on optical observation [10]. Because of the difficulty of measurement, few studies have focused on the crystal ultrastructure of eggshells. X-ray crystallography has shown that calcium carbonate in avian eggshell is in the form of calcite crystals [11, 12]. The microstructural characteristics influenced by the intricate interlacing of the crystal units have been reported to vary significantly from one eggshell to another, even within the same species [13–15]. The eggshell consists of ceramic material whose mechanical properties are determined by its microstructure [16]. Chicken eggshells consisting of highly-oriented crystals of abnormal sizes have been reported to be generally weaker than those consisting of smaller and less well-oriented crystals [14, 17]. A comparison of eggshells at different ages showed that aged hens produced a greater variation in grain morphology and crystallographic texture than young hens [17]. The crystallographic texture of eggshells is also correlated with the thickness and strength of the shell. The crystal size and orientation has been found to be strongly positively correlated (*r* = 0.65) with the thickness of the eggshell [18]. The degree of orientation of crystals has been found to explain approximately 40 % of the variance in eggshell strength [17]. All these studies have indicated that the ultrastructure of eggshell was the key factor affecting eggshell quality.

Eggshell texture and ultrastructure are regulated by organic matrix proteins, which control the mineralization process and influence the biomechanical properties of the eggshell [19]. Association studies between polymorphisms of genes encoding shell proteins and shell characteristics have revealed that certain alleles were correlated with the mechanical properties of the eggshell [20, 21]. Ovalbumin and ovo-transferrin (*LTF*) markers were found to be associated with crystal size, while ovocleidin-116 and ovocalyxin-32 (*RARRES1*) markers were associated with crystal orientation [18]. Because the crystal is the basic component of construction of the eggshell, the alleles of matrix proteins and genes known to be involved in eggshell formation have also been found to be associated with crystal size and orientation [7, 18, 21]. Therefore, genetic selection of hens with suitable eggshell ultrastructure properties is important for improving eggshell quality. However, the genetic mechanism of the eggshell crystal ultrastructure is still unclear.

In the present study, the crystal structure of chicken eggshells was determined using X-ray crystallography. To study the genetic basis of biomineralization, a genome-wide association study (GWAS) was performed in the designated F2 population by using a high-density 600k SNP chip for chickens. We aimed to explore the genetic architecture of eggshell crystal structure to identify candidate genes, which may be valuable for the genetic improvement of eggshell quality traits.

## Results

### Phenotypic description and genetic parameters

The descriptive statistics for nine reflection peaks (A1-A9), total integral intensity (TA) and degree of orientation (OD) were shown in Table 1. The estimates of SNP-based heritability, the genetic and phenotypic correlations between eggshell crystal structure traits were shown in Table 2. The individual integral intensity exhibited higher phenotypic variation (11.71–21.23 %) than that of TA (9.40 %). Thus, the TA could provide a good estimate of the size of the eggshell crystals. The SNP-based heritability (h^2^_snp_) of TA was 0.23. The phenotypic correlations between every individual integral intensity (A1-A9) were low (*r*=-0.15–0.35). The absolute values of the genetic correlations were all higher than those of the phenotypic correlations. The variation in OD was higher (23.72 %) than that of the other traits and the value of h^2^_snp_ was low (0.06).

**Table 1:** Descriptive statistics for individual integral intensity, total integral intensity and degree of orientation

Traits	N	Mean	SD	CV (%)	Min	Max
A1	839	25335.41	4813.91	19.00	12953.58	38929.12
A2	839	239384.50	49882.98	20.84	104335.20	372372.10
A3	839	50264.81	10673.14	21.23	24546.91	80485.42
A4	839	71896.02	10669.69	14.84	44550.43	100413.00
A5	839	70718.49	13935.86	19.71	36597.64	108482.90
A6	839	131559.70	17180.31	13.06	83325.08	180757.50
A7	839	57379.84	7497.32	13.07	37028.29	78801.67
A8	839	38877.79	4554.47	11.71	26827.77	51180.12
A9	839	40565.02	5966.54	14.71	24762.44	56568.73
TA	837	726470.70	68300.65	9.40	549996.40	906125.60
OD	822	1.71	0.41	23.72	1.00	2.86

Abbreviations: N = number of samples; Mean = arithmetic mean; SD = standard deviation; CV = coefficient of variance; Min = minimum; Max = maximum; A1-A9 = integrate intensity of peaks of No. 1–9; TA = total intensity of all 9 peaks; OD = degree of orientation

**Table 2:** Summary of genetic analysis between individual integral intensity, total integral intensity and degree of orientation

Traits	A1	A2	A3	A4	A5	A6	A7	A8	A9	TA	OD
A1	**0.05** **(0.05)**	0.22 (0.52)	0.94 (0.5)	0.63 (0.48)	NA	0.45 (0.48)	0.87 (0.42)	1.00 (0.66)	NA	0.87 (0.44)	0.41 (0.65)
A2	0.02 (0.03)	**0.10** **(0.05)**	-0.50 (0.47)	0.46 (0.27)	0.17 (0.53)	1.00 (0.18)	0.59 (0.3)	0.73 (0.23)	0.36 (0.44)	0.90 (0.07)	NA
A3	0.09** (0.03)	-0.05 (0.03)	**0.06** **(0.05)**	0.68 (0.25)	0.83 (0.39)	-0.43 (0.38)	0.84 (0.22)	0.51 (0.36)	0.27 (0.53)	0.17 (0.33)	1.00 (0.47)
A4	0.07 (0.03)	0.07** (0.03)	0.26** (0.03)	**0.21** **(0.06)**	0.84 (0.32)	0.07 (0.24)	0.80 (0.15)	0.74 (0.18)	0.45 (0.31)	0.71 (0.14)	0.56 (0.24)
A5	0.02 (0.03)	0 (0.03)	0.27** (0.03)	0.17** (0.03)	**0.05** **(0.05)**	0.64 (0.53)	0.63 (0.35)	0.83 (0.34)	1.00 (0.84)	0.85 (0.38)	0.64 (0.5)
A6	0.07* (0.03)	0.41** (0.03)	-0.15** (0.03)	0 (0.03)	-0.06 (0.03)	**0.15** **(0.05)**	0.38 (0.25)	0.74 (0.21)	0.46 (0.37)	0.88 (0.11)	-0.88 (0.29)
A7	0.16** (0.03)	-0.04 (0.03)	0.35** (0.03)	0.32** (0.03)	0.32** (0.03)	-0.11** (0.03)	**0.24** **(0.06)**	0.75 (0.18)	0.61 (0.31)	0.79 (0.15)	0.44 (0.3)
A8	0.11* (0.03)	0.29** (0.03)	0.20** (0.03)	0.29** (0.03)	0.20** (0.03)	0.23** (0.03)	0.28** (0.03)	**0.15** **(0.05)**	0.94 (0.34)	0.94 (0.10)	0.06 (0.38)
A9	0.08* (0.03)	0.10* (0.03)	0.14** (0.03)	0.20** (0.03)	0.07* (0.03)	0.13* (0.03)	0.26** (0.03)	0.31** (0.03)	**0.07** **(0.05)**	0.61 (0.31)	0.37 (0.51)
TA	0.15** (0.03)	0.84** (0.01)	0.25** (0.03)	0.34** (0.03)	0.31** (0.03)	0.52** (0.02)	0.26** (0.03)	0.50** (0.02)	0.31** (0.03)	**0.23** **(0.06)**	-0.12 (0.30)
OD	0.04 (0.03)	-0.78** (0.02)	0.18** (0.03)	0.52** (0.02)	0.10** (0.03)	-0.34** (0.03)	0.23** (0.03)	-0.04 (0.03)	0.03 (0.03)	-0.48** (0.02)	**0.06** **(0.05)**

Lower triangle: phenotypic correlations; Diagonal: heritability estimates based on genome; Upper triangle: genetic correlations; Standard errors of the estimates are in parentheses; A1-A9 = integrate intensity of peaks of No. 1–9; TA = total intensity of all 9 peaks; OD = degree of orientation; ^*^Significant linkage at *P* < 0.05. ^**^Significant linkage at *P* < 0.01. NA: the genetic correlation value did not converge when we calculated the genetic correlation by the GCTA

**Fig. 1:**
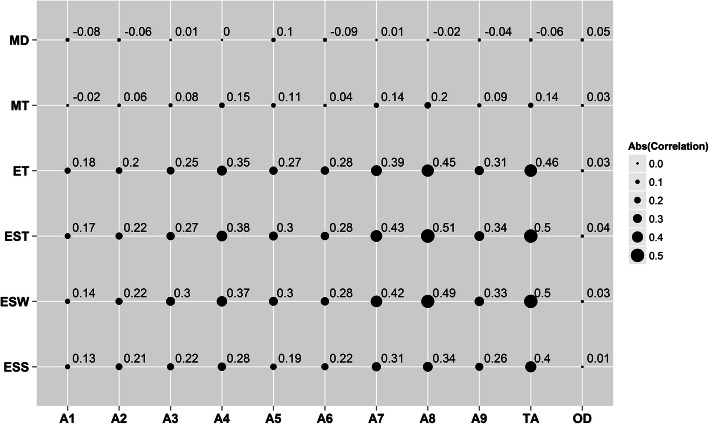
The phenotypic correlations between the crystal structure and eggshell quality. Abbreviations: A1-A9 = integrate intensity of peaks of No. 1–9; TA = total intensity of all 9 peaks; OD = degree of orientation; ESS = eggshell strength; ESW = eggshell weight; EST = eggshell thickness; ET = effective layer thickness; MT = mammillary layer thickness; MD = mammillary density. Abs (Correlation) = Absolute Correlation

In a previous study, we also measured the ultrastructure of these eggshells [22]. The phenotypic correlations between the crystal structure and eggshell ultrastructure are shown in Fig. 1. TA was positively correlated with eggshell strength (ESS), eggshell thickness (EST), eggshell weight (ESW), mammillary layer thickness (MT) and effective layer thickness (ET) (*r *= 0.14–0.50), but OD was not significantly correlated with these eggshell quality traits (*p* < 0.05). There was a high genetic correlation between the total peak area intensity and eggshell ultrastructure (*r* = 0.52), and a medium genetic correlation between the OD and the eggshell ultrastructure (*r* = 0.31) (Table 3). However, the genetic correlation of crystal structure with the eggshell ultrastructure all exhibited a large standard error (Table 3).

**Table 3:** The genetic correlations between the crystal structure and eggshell quality traits

Traits	MD	MT	ET	EST	ESW	ESS
A1	-0.38(0.39)	0.01(0.39)	0.66(0.36)	0.53(0.32)	0.77(0.32)	0.95(0.55)
A2	-0.82(0.19)	0.62(0.23)	0.45(0.22)	0.62(0.19)	0.46(0.19)	0.13(0.25)
A3	0.22(0.35)	-0.08(0.33)	0.79(0.20)	0.69(0.23)	0.63(0.20)	0.48(0.28)
A4	0.01(0.21)	0.14(0.20)	0.82(0.11)	0.73(0.11)	0.63(0.11)	0.55(0.14)
A5	-0.33(0.33)	0.57(0.37)	0.82(0.28)	0.92(0.30)	0.80(0.20)	0.27(0.29)
A6	-0.30(0.24)	0.30(0.23)	0.64(0.15)	0.67(0.14)	0.45(0.16)	0.44(0.18)
A7	-0.21(0.20)	0.16(0.21)	0.97(0.10)	0.97(0.07)	0.90(0.08)	0.80(0.12)
A8	-0.24(0.22)	0.46(0.20)	0.99(0.09)	0.99(0.08)	0.76(0.11)	0.45(0.18)
A9	-0.56(0.28)	0.21(0.29)	0.68(0.24)	0.60(0.24)	0.49(0.23)	0.50(0.25)
TA	-0.61(0.17)	0.53(0.17)	0.83(0.09)	0.88(0.07)	0.75(0.09)	0.52(0.14)
OD	0.87(0.31)	-0.42(0.30)	0.48(0.29)	0.39(0.28)	0.31(0.26)	0.65(0.28)

Standard errors of the estimates are in parentheses; A1-A9 = integrate intensity of peaks of No. 1–9; TA = total intensity of all 9 peaks; OD = degree of orientation; ESS = eggshell strength; ESW = eggshell weight; EST = eggshell thickness; ET = effective layer thickness; MD = mammillary density; MT = mammillary layer thickness

### Genome-wide association study (GWAS)

**Fig. 2:**
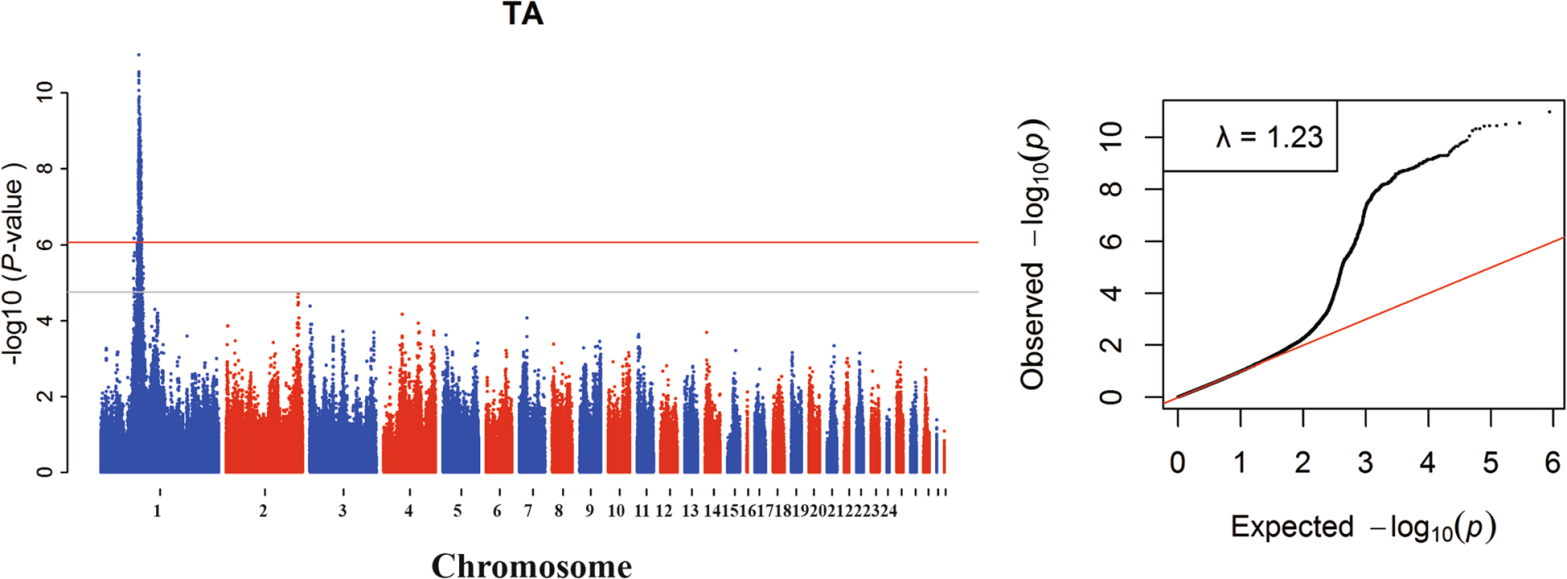
Manhattan plots (left) and quantile–quantile plots (right) of the observed P-values for TA. The Manhattan plots indicate -log_10_ (observed P-values) for genome-wide SNPs (y-axis) plotted against their respective positions on each chromosome (x-axis), and the horizontal gray and red lines depict the genome-wide suggestive (1.73 × 10^− 5^) and significant (8.64 × 10^− 7^) thresholds, respectively. For quantile-quantile plots, the x-axis shows the expected -log_10_-transformed P-values, and the y-axis represents the observed -log_10_-transformed P-values. The genomic inflation factors (λ) are shown on the top left in the QQ plot. TA represents the total integral intensity of all peaks

The association analyses revealed a total of 621 genome-wide significant SNPs associated with TA (Additional file 1: Table S2). No genome-wide significant locus was associated with OD. In addition, 1096 and 1 SNPs exhibited associations with TA and OD under suggestive levels of significance, respectively. The Manhattan and quantile-quantile (QQ) plots in Fig. 2 showed a global view of the *P*-values for all SNPs affecting the ultrastructure of the eggshell crystals. All significant SNPs associated with TA were in a 13.5-Mb region between 55.6 and 69.1 Mb on chromosome 1 (GGA1).

**Fig. 3:**
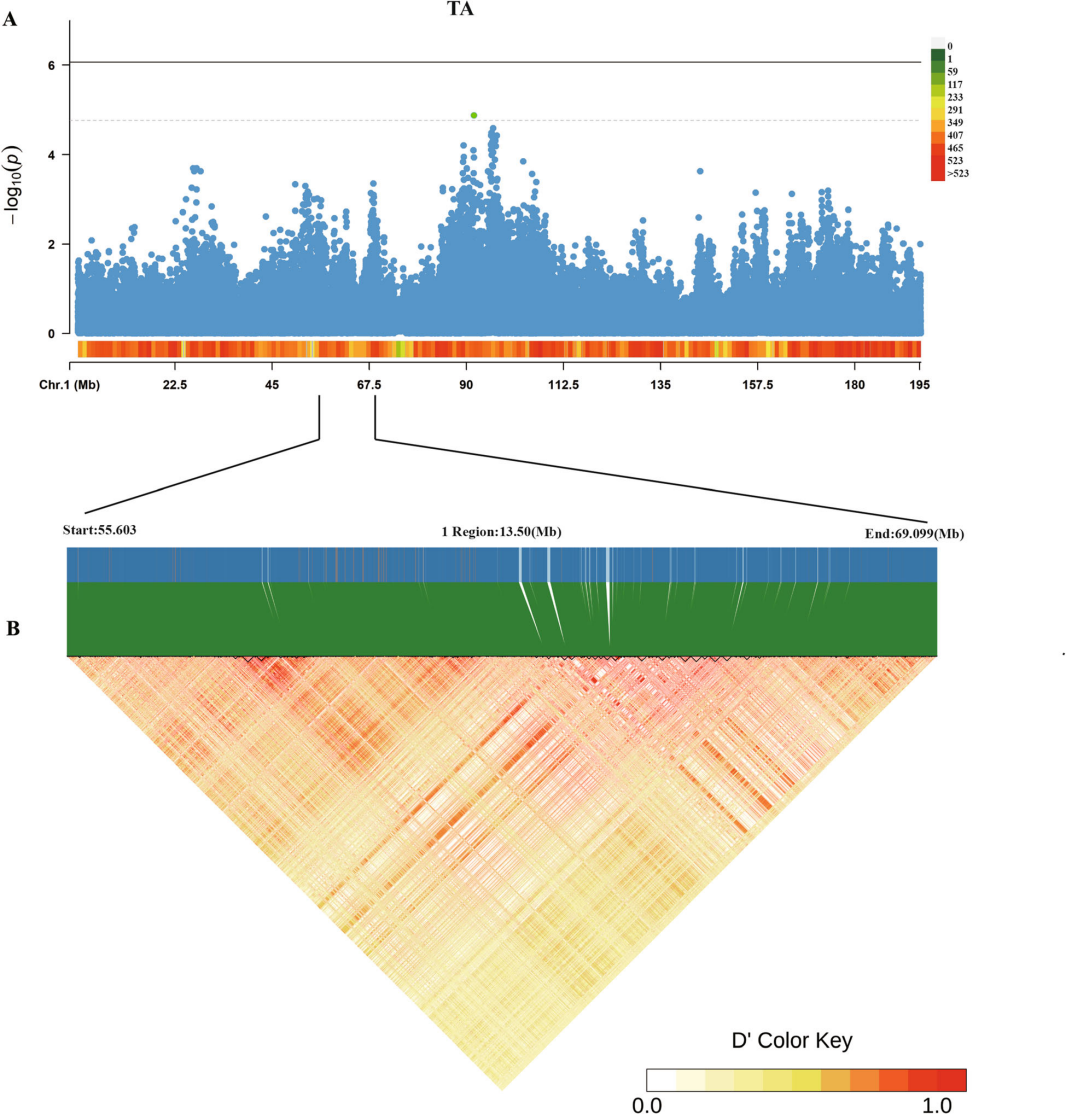
Conditional association analyses (**A**) and linkage disequilibrium (LD) analysis (**B**) for TA. The genotype of rs315989578 was added to the multivariate model as a covariate for the conditional analysis. The LD plot of significant SNPs with the total intensity of all peaks in the region ranged from 55.6 to 69.1 Mb. Haplotypes are indicated by sample symbols connected by a solid line. The color represents Hedrick’s multiallelic D’, which indicates the degree of LD between two blocks. TA represents the total integral intensity of all peaks

To identify independent SNPs, a further series stepwise conditional analyses were performed and the locus rs315989578, that was found to be significantly associated with TA was fitted into the model to examine these associations. The levels of significance for all loci around the most significant SNP decreased substantially (Fig. 3A) during the stepwise conditional Genome-wide Association (GWA) analysis, with no other independent loci being detected. The Fig. 3B illustrated the linkage disequilibrium between the regions associated with TA traits. A region showing a relatively strong linkage disequilibrium was in chr1: 55.6 Mb and 69.1 Mb. We also conducted a GWAS analysis on A1-A9 (Additional file 2: Table S1). The result showed that no significant SNPs were associated with the A1, A2, A3, A4, A5, A7 and A8. 163 and 330 genome-wide significant SNPs were associated with the A6 and A9, respectively. These significant SNPs almost coincided with the significant SNPs for TA.

### SNP annotation and promising genes associated with eggshell ultrastructure

The annotation of significant SNPs using the Variant Effect Predictor (VEP) tool supplied by Ensembl could help identify promising genes associated with eggshell crystal structure. Detailed information about the genes is summarized in Additional file 1: Table S2. In total, 74 potential candidate genes all with significant quantitative trait loci (QTLs) were identified. The results of enrichment analysis were listed in Additional file 1: Table S3. We found that six genes were significantly enriched in the blood circulation (GO:0008015): *CACNA1C* (voltage-dependent L-type calcium channel subunit alpha-1 C), *IAPP* (islet amyloid polypeptide), *ITPR2* (inositol 1,4,5-trisphosphate receptor type 2), *PLCZ1* (1-phosphatidylinositol 4,5-bisphosphate phosphodiesterase zeta-1), *ABCC9* (ATP-binding cassette sub-family C member 9) and *KCNJ8* (ATP-sensitive inward rectifier potassium channel 8). Among them, the *CACNA1C, IAPP, ITPR2* and *PLCZ1* also played considerable roles in the biological process of regulating cytosolic calcium ion concentration. We also found 26 genes that were regulated by the octamer transcription factor 1 (Oct-1). However, no information on the interaction network is available for these candidate genes.

### SNP contribution to phenotypic variation

**Fig. 4:**
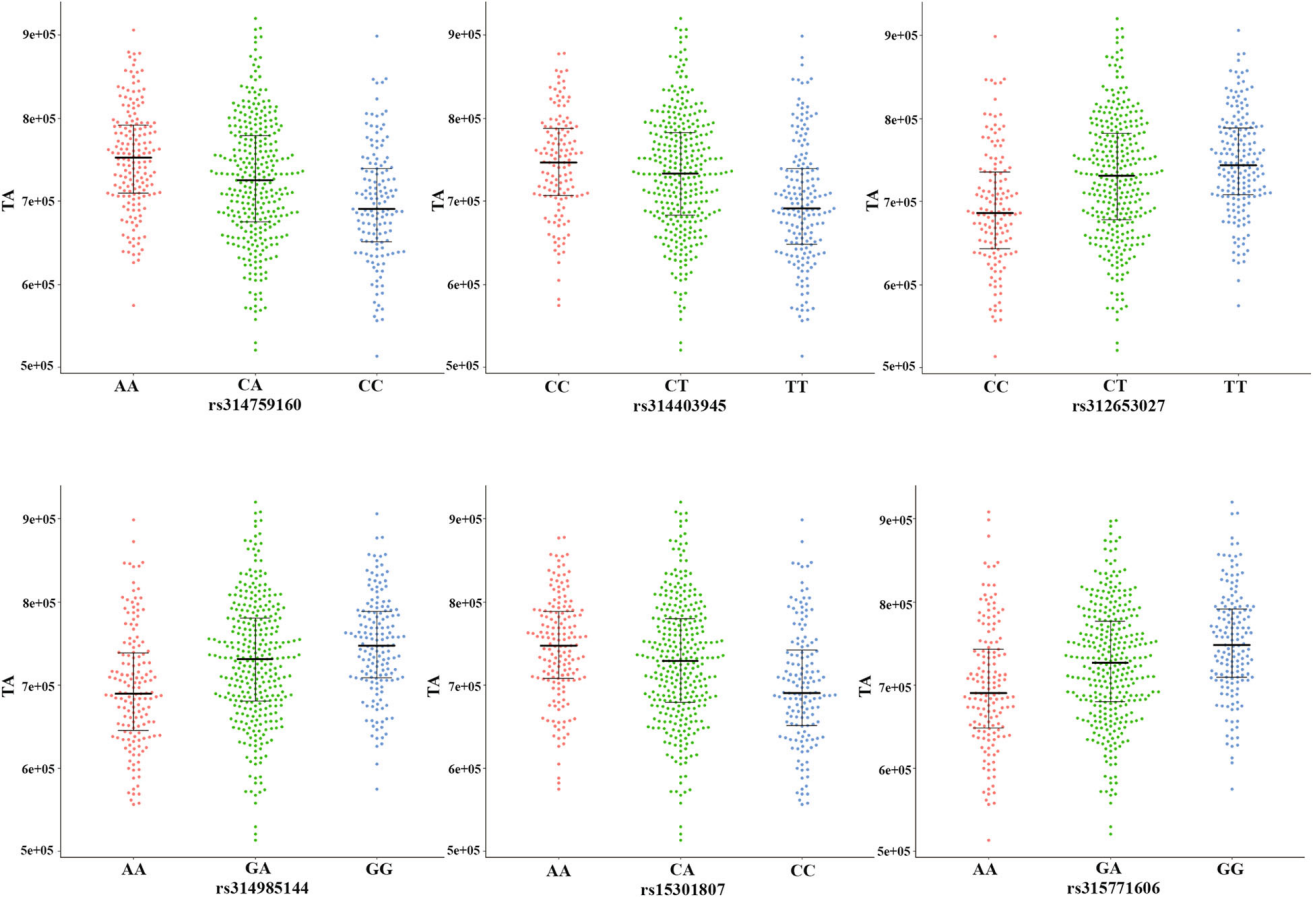
Boxplots of the SNP effects on the TA. The figure shows the genotypes of six representative SNPs (x-axis) versus the phenotypic values of the corresponding traits (y-axis). TA represents the total integral intensity of all peaks

Table 4 showed six meaningful loci extracted which reached genome-wide significance in the univariate GWAS for analysis: rs314759160, rs314985144, rs314403945, rs15301807, rs315771606 and rs312653027 in *PLCZ1*, *ABCC9*, *ITPR2*, *KCNJ8*, *CACNA1C* and *IAPP*, respectively. These six SNPs exerted contributions of between 3.93 % and 5.90 % to the phenotypic variation in TA. Notably, rs314759160 exhibited the largest allelic substitution effect on TA with the substitution of one copy of the effect allele at the rs314759160 site causing a 0.345 SD/allele decrease in TA. The phenotypic differences between the genotypes of those SNPs were shown in boxplots (Fig. 4), revealing that homozygotes of the effect allele and of the alternative alleles possessed the lowest and highest phenotypic values, respectively, whereas the values for the heterozygotes were intermediate. Data also showed that the most significant QTL associated with TA was the rs315989578 at the position 63.8 Mb on chromosome 1 (*p*-value 1.01e-11). However, this SNP located in the intergenic region, explained 6.45 % of the phenotypic variation (Additional file 1: Table S2).

**Table 4:** Contributions of six SNPs in genes potentially related to the total integral intensity

SNP	Gene	Consequence	Chr.	Position (bp)	EA/AA	P	MAF	Beta (SE)	CPV (%)
rs314759160	*PLCZ1*	intron_variant	1	64,223,356	A/C	4.96E-10	0.454	-0.345(-0.055)	5.90
rs314403945	*ITPR2*	intron_variant	1	67,660,408	T/C	9.18E-09	0.475	0.320(-0.055)	5.11
rs312653027	*IAPP*	downstream_gene_variant	1	65,271,009	C/T	5.42E-09	0.443	-0.391(-0.054)	5.02
rs314985144	*ABCC9*	intron_variant	1	66,908,211	G/A	2.75E-08	0.468	-0.305(-0.054)	4.63
rs15301807	*KCNJ8*	intron_variant	1	66,959,507	A/C	3.52E-07	0.470	-0.285(-0.056)	4.05
rs315771606	*CACNA1C*	intron_variant	1	61,199,051	G/A	5.13E-07	0.468	-0.281(-0.056)	3.93

Abbreviations: EA/AA: effect allele (minor allele)/alternative allele (major allele); EA = effect allele (minor allele); AA = alternative allele (major allele); MAF = minor allele frequency; CPV = contribution to phenotypic variance. Beta is the estimated allelic substitution effect per copy of the effect allele (EA) based on an inverse-normal transformed scale under an additive model, expressed in SD unit/allele; SE = standard error of the beta

## Discussion

The eggshell of chicken is a bio-ceramic material comprising columnar calcite (CaCO_3_) crystals and organic proteinaceous matrix [7]. Previous studies have found that the size and orientation of calcium carbonate crystals influenced the eggshell quality [17, 18]. In the present study, we used X-ray diffraction to measure the traits of calcium carbonate crystals in the eggshell. This method was based on the analysis of X-ray diffraction patterns formed by intact eggshells, with the intensity of the spots displayed in these patterns being directly related to the size of crystals in the eggshells [12]. Our results showed that the ESS, ESW, EST, ET and MT increased as the size of the crystals increased (Fig. 1), which was consistent with previous studies [18, 21]. We speculated that in eggshells with larger crystals a faster crystal growth rate had occurred, causing the deposition of more calcites in the eggshell and thus increasing its strength, weight and thickness [23]. Moreover, the palisade layer was mainly composed of packed calcite crystals, being governed by the geometry of the nucleation sites, crystal growth rate and interactions between the growing crystals [24]. The thickness and strength of the eggshell would increase as the size of the calcite crystal increases[18]. Interestingly, our results have shown that the TA was highly negatively correlated with the OD (*r* = − 0.48, Table 2). This indicated that the crystal structure of the larger crystals was more randomly oriented, which was consistent with a previous study [18]. Table 3 showed that TA was highly genetically correlated with the microstructure traits ET, MT, ESS and EST, results consistent with those of Dunn’s study [18]. We also noted that the genetic correlations were high between the A7, A8, ET and EST. However, this high correlation has not been reported in other studies. X-ray diffraction studies have showed that the ET was oriented to a few crystal planes [5]. So, we hypothesized that the arrangement of some specific crystal planes (such as A7 and A8) may have an important effect on the eggshell structure. However, further study is needed for the crystallographic structure mechanism. In the present study, OD was not correlated with the quality of the chicken eggshells. The estimated heritability of OD (*h*^2^ = 0.06) was also low with a large estimation error, but was smaller than previous estimates [18], partly because of the higher variation coefficient for OD (Table 2). The genetic correlations between OD and ET in the present study were high (*r* = 0.48) (Table 3), and Dunn et al. also found a similar genetic correlation (*r* = 0.32) between OD and ET[18], but the estimation errors were large in both studies. To determine whether this estimate is reliable, our experiments will need to be repeated with a larger sample. Therefore, further studies are needed to explore the mechanical aspects of crystallographic texture.

The eggshell is composed of four layers, with ET accounting for more than 70 % of the total thickness [25]. Therefore, changes in the effective layer of eggshells can significantly affect their properties [26]. In a previous study, the phenotypic correlation between the EST and ET of eggshells was 0.92, while the genetic correlation was 0.96 [22]. The association analysis of QTLs in the region between 57.3 and 71.4 Mb of GGA1 was significantly associated with ESS, ESW and EST [27]. We have also identified a 9-Mb QTL (GGA1: 59.4–68.5 Mb) in this region which was associated with the ET [22]. These results have indicated that this region of chromosome 1 had a great influence on the microstructure of eggshells, but the details are unclear. Interestingly, the present study has found that this region was also closely related to the TA. One reason may be that there was a strong correlation (*r* = 0.5) between the ET and TA (Fig. 1). However, no QTLs were identified as being associated with crystal growth orientation. We speculated that the size of the crystal was the dominant influence on the structure of the eggshell, which was independent of the orientation of crystal growth. Our results have also shown that GGA1 was closely related to the ultrastructure of the chicken eggshell.

The formation of eggshells is mainly achieved by biomineralization in the chicken uterus [16, 28, 29]. Two systems have been proposed as playing an important role in the biomineralization of eggshells: the ion transport system and the matrix protein system [30]. The ion transport system provides sufficient ions for eggshell formation and matrix proteins play a regulatory role in the process of eggshell mineralization [31, 32]. In a previous study, *ABCC9*, *ITPR2*, *KCNJ8* and *WNK1* have been mapped as being associated with ET and EST [22], and all these genes are also involved in ion transport with their expression detected in the chicken uterus. Many studies have also reported that the eggshell matrix proteins were associated with EST [7, 21]. Genetic associations for crystal measurements showed that OVAL and ovo-transferrin were associated with TA, while OC116 and OCX-32 were associated with OD [18]. In the present study, we have identified 76 candidate genes including *ABCC9*, *ITPR2*, *KCNJ8* as being associated with crystal size and six genes with the process of blood circulation (Additional file 1: Table S3). *ABCC9* and *KCNJ8* encoded membrane-associated receptors that combined with the potassium channel have been reported to become smooth muscle ATP-sensitive potassium channels in the heart [33]. Inositol 1,4,5 trisphosphate receptors (*ITPR*s), a family of endoplasmic reticulum Ca^2+^ channels, are essential for controlling intracellular Ca^2+^ levels in virtually every type of mammalian cell [34]. *ITPR2* has been found to be highly expressed in cardiomyocytes and to be associated with human heart failure [35, 36]. As important ion transmembrane channels, these genes are reported to play a vital role in cardiac contraction [37]. It has been reported that the calcium required for eggshell calcification mainly depends on blood circulation, and more efficient calcium transport could promote the calcification of eggshells [38]. Our results thus suggested that the strength of cardiac contractility may affect the mineralization of eggshells.

Several genes related to intracellular calcium concentration were also identified. *ITPR2* was also directly involved in the process of Ca^2+^ signal transduction and released during bone growth [39]. Ca^2+^ oscillation is mediated by the inositol 1,4,5-trisphosphate receptors 2 and 3 (*ITPR2* and *ITPR3*) in the osteoclast differentiation [40]. Except for previously reported major candidate genes, we have also identified several new genes associated with crystal size, *CACNA1C*, *IAPP* and *PLCZ1*, which influence the function of regulating cytosolic calcium ion concentration. *CACNA1C* encoded the protein, voltage-dependent L-type calcium channel subunit alpha-1 C, which is essential for normal blood pressure regulation through the contraction of arterial smooth muscle cells [41]. *CACNA1C* is highly expressed in the brain, heart, jejunum, ovary, pancreatic beta-cells and vascular smooth muscle [41–43]. Ca^2+^ influx through *CACNA1C* has also been reported to activate osteogenic transcriptional programs and promote mineralization [44]. We therefore propose that *CACNA1C* might play an important role in regulating the concentration of calcium in uterine fluid. Amylin is encoded by *IAPP* and is a member of the calcitonin family of hormones that is co-secreted with insulin by the pancreatic beta cells [45]. Amylin has been reported to use the calcitonin receptor to inhibit osteoclastogenesis in vivo [45]. There was no direct evidence that *PLCZ1* participated in the release of calcium but it has been reported that it indirectly affected the transmembrane transport of calcium [46, 47]. Interestingly, we also found that 22 candidate genes, including *ITPR2*, *PLCZ1* and *CACNA1C*, were regulated by the transcription factor, Oct-1, which acted as downstream of notch signaling during radial glia formation. Oct-1 has been reported as forming a novel complex with the transcription factor (Runx2) to regulate the expression of the mammary gland-specific gene, beta-casein. Runx2 is essential for expressing several bone-specific genes and is primarily considered as a master regulator of bone development [48]. This suggested that Oct-1 might also be related to the development of bone. The present study has shown that several genes related to bone growth were significantly associated with the strength of eggshell crystals. Except for eggshell formation, the biomineralization process in birds occurs in the bone calcification [49]. Some eggshell matrix proteins such as OC-116 have been found to be expressed in bone [50], which indicated that there may be similar biological pathways in the two mineralization processes. Our results further support this hypothesis. It is also worth noting that no known eggshell matrix protein was found in these candidate genes, because some matrix proteins such as ovocleidin*-*17 have not yet been annotated in the reference genome. It will thus be necessary to obtain more information on the crystallization system for eggshells.

## Conclusions

The integral intensity and degree of orientation of eggshell crystal were determined, then used for a GWA analysis with a 600 K high-density SNP array. The result showed that 621 genome-wide significant SNPs ranging from 55.6 to 69.1 Mb in GGA1 were associated with TA. Ultimately, six genes, *PLCZ1*, *ABCC9*, *ITPR2*, *KCNJ8*, *CACNA1C* and *IAPP*, were considered as promising genes that were implicated in the integral intensity of eggshell crystals and could improve eggshell quality. The present study has revealed the genetic basis of the crystallographic ultrastructure of eggshells and improved our understanding of its mechanical properties. These new findings will also provide a useful theoretical basis on biomineralization in chicken eggshells.

## Methods and materials

### Experimental population

White Leghorn (WL) and Dongxiang (DX) chickens, representing a standard breed and a Chinese indigenous strain, respectively, were used to construct an F2 resource population for the present study. Six WL and six DX males were mated with 133 DX and 80 WL females, respectively. Then, 25 cocks and 407 hens from the WL/DX cross and 24 cocks and 235 hens from DX/WL cross in the F1 generation were selected to produce the F2 generation. A total of 3749 chickens from the F2 population were reared in individual cages with ad libitum access to feed. Finally, 839 hens from 49 half-sib families and 365 full-sib families with sufficient phenotypic and pedigree information were chosen for SNP genotyping. All the experimental animals were hens and came from the same batch.

### Phenotypic measurements

Eggs were collected from hens at 66 weeks of age on three successive days to ensure at least one egg per hen. The egg contents were removed then the eggshells were cleaned with tap water and dried naturally at room temperature. Pieces (approximately 3 × 5 mm) of eggshell were taken at the equator. The shells were measured using X-ray diffraction (R-Axis Spider XRD, Rigaku Corp., Tokyo, Japan). The measurement conditions were as follows: Mo Kα (λ = 0.71075 Å), 50 KV, 80 mA, a pin-hole collimator (0.8 mm in diameter) and an exposure time of 30 s per frame. The samples were mounted with the inner surface of the eggshell facing the incident X-ray beam. The beam passed through the sample and the transmission diffraction pattern was recorded on the detector. The software THCLXPD attached to the XRD was used to analyze the diffraction patterns by measuring the intensity of the reflection spots.

The integrated intensities were determined from each eggshell sample using nine different rings. These rings were oriented with a set of (hkl) crystallographic planes: 113, 104, 012, 110, 202, 018, 122, 208 and 300. The data from the nine reflection peaks (A1-A9) were added to give the TA to lower the data variability. The crystal size was estimated from the intensity of the reflection spots [51]. To determine the OD, a calcite powder standard (Joint Committee on Powder Diffraction File, PDF 05-0586) having a completely random orientation was used as a reference pattern for the crystals. The ratio between the integrated intensities of the calcite reflections from the eggshell and those of the standard sample (PDF 05-0586) provided a measure of the relative orientation of the eggshell crystals [9]. In the present study, the strongest integrated intensities of the standard sample (PDF 05-0586) were 113 and 104 (Additional file 1: Table S1), indicating that the crystals were preferentially oriented to these planes. The ratio (R_std_) of the calcite powder standard sample (A_113_/A_104_) was 0.18. Thus, the OD of eggshell crystals was calculated using Eq. (1):

1 $$\text{OD=}\frac{\text{R}}{{\text{R}}_{\text{std}}}\text{=}\frac{\frac{{\text{A}}_{\text{sample}}\text{(113)}}{{\text{A}}_{\text{sample}}\text{(104)}}}{\text{0.18}}$$

The value of OD represented the degree of the preferential orientation of the crystals. For a value of one, the sample consists of randomly oriented crystals and for a value > 1, the sample consists of highly-oriented crystals [17].

The data on eggshell quality traits (ESS, EST, ESW, MT and ET) from our previous publications was used in the correlation analysis[22, 52]. The descriptive statistics of all the phenotypic data were calculated using R (version 3.3.1) (https://www.rproject.org/).

### Genotyping and quality control (QC)

All blood samples were collected from brachial veins of chickens by standard venipuncture. Genomic DNA was extracted from whole blood samples using standard phenol-chloroform method and the 839 chickens were genotyped using the Affymetrix 600 K chicken SNP chip (Affymetrix Inc. Santa Clara, CA, USA). Array Power Tools (APT) (version 1.16.0; Affymetrix Inc) software was used for quality control and genotype calling. SNPs with a minor allele frequency of < 5 % and a Hardy-Weinberg equilibrium test with *P* < 1 × 10^− 6^ were removed using the PLINK version 1.90 package. The BEAGLE version 4.0 procedure [53] was used to impute sporadic missing genotypes, and SNPs with an imputation quality score of *R*^2^ > 0.5 were retained for the next analysis step. A total of 839 samples and 435,867 SNPs finally remained for the subsequent GWAS.

### GWA analysis

The loci-based analysis was performed using the generalized linear mixed model implemented in GEMMA [54], where the kinship matrix is calculated using the standardization method. The mixed model was based mainly on the additive effect of sites:

2 $$\text{Y}\text{=1}\mathrm{\mu}\text{+}\text{Xb}\text{+}\text{Zu}\text{+}\text{S}{\mathrm{\alpha}}\text{+}\text{e}$$

Where: Y is the vector phenotypes of eggshell; µ the overall mean; X the covariance matrix (containing the first ten PCA principal components obtained from analysis of the population substructure); b the estimator vector of fixed effects; Z is an incidence matrix associating u with y. u the additive polygenic effect (0, GV_g_), with G the genomic kinship matrix, and the additive effect variance Vg; S the design matrix containing the corresponding SNP sites; α the substitution effect size corresponding to each site; and e the vector of random residual effects (N (0, IV_e_)), with I the identity matrix and the residual variance (V_e_).

The genomic inflation factor (λ) was calculated using the R package, qqman (Version 0.1.2) [55]. Multiple test thresholds were calculated using the simpleM method [56]. Stepwise conditional analyses were conducted using the generalized linear mixed model of the R package with the strongest SNP identified being added at each step as a covariate. A total of 58,358 valid inspections was obtained. The genome-wide significance level was 8.57 × 10^− 7^ (0.05/58,358), with a suggestive significance level of 1.71 × 10^− 5^ (1.00/58,358). Principal component analysis was performed using the EIGENSOFT software (v.2.04) with the results showing that there was stratification in the experimental population. In order to correct the population stratification, we selected the top ten PCA [57] components as covariates in the GWAS analysis.

The SNP-based heritability of eggshell crystal traits, calculated using the residual maximum likelihood (REML) method in GCTA (version 1.24) [58], was estimated from the same mixed model as used in the GWAS analysis. The genetic correlations were estimated from a standard bivariate linear mixed model [59] using the Bivariate GREML analysis of GCTA [60]. We used Visscher’s method [61] to calculate the power of genetic correlation. We calculated the phenotypic variance contribution (CPV) of those genome-wide significant SNPs based on the equation $\text{2}\text{pq}{\mathrm{\beta}}^{\text{2}}/{\mathrm{\sigma}}^{\text{2}}$, with allele frequencies p and q, β was corresponding effect size of SNP identified in association study, ${\mathrm{\sigma}}^{\text{2}}$ was the phenotypic variance [62].

### Linkage disequilibrium analysis and gene identification

We performed a series of linkage disequilibrium (LD) analyses to characterize the causative SNPs within strong LD regions by applying the solid spine algorithm in Haploview software version 4.2 [63]. The significant SNPs were functionally annotated, then the candidate genes related to the significant SNPs or genomic regions were identified using the Variant Effect Predictor [64]. The Biomart tools were supported by Ensembl based on the Galgal6 assembly.

### Functional annotation

Gene Ontology annotation and KEGG pathway were completed using the Metascape website to assign genes with corresponding terms [65, 66]. The identities of all the candidate genes were converted into those of the homologous genes in humans.

## Supplementary information

**Additional file 1.**

**Additional file 2.**

## Data Availability

The datasets generated for this study can be found in figshare repository, 10.6084/m9.figshare.16627099.v1 .

## Abbreviations

TA: total integral intensity
OD: degree of orientation
ESS: eggshell strength
EST: eggshell thickness
ESW: eggshell weight
MT: mammillary layer thickness
ET: effective layer thickness
GWAS: Genome-wide association study
GWA: Genome-wide association
SNP: Single nucleotide polymorphism
QTL: Quantitative trait loci
LD: Linkage disequilibrium
REML: Restricted maximum likelihood
MAF: Minor allele frequency
HWE: Hardy-Weinberg equilibrium
PCA: Principal component analysis
VEP: Variant effect predictor
